# Classification of retinopathy of prematurity: from then till now

**Published:** 2017-11-11

**Authors:** Komal Agarwal, Subhadra Jalali

**Affiliations:** 1 Consultant: Srimati Kannuri Santhamma Centre for vitreoretinal diseases and Jasti V Ramanamma Childrens' Eye Care centre Kallam Anji Reddy Campus, L V Prasad Eye Institute, Hyderabad, India.; 2 Deputy Director: Newborn Eye Health Alliance (NEHA) and Director, Quality: LV Prasad Eye Institute, Hyderabad, India.

**The complex and variably progressive nature of ROP warrants a robust description of the disease and its classification into various severities which helps the clinicians to properly document, prognosticate and treat the disease.**

The first case of ROP was described in the 1940s, very soon after the first incubators were set up in childrens' hospitals.^1^ At birth, most preterm babies would have an immature retina, defined as retinal vessels not reaching the ora serrate (end of the retinal tissue) and caliber of vessels is normal with vessels showing a dichotomous branching pattern (Figures 1a,1b). After birth in 2–3 weeks, ROP starts manifesting and can be seen on fundoscopy. ROP is a rapidly changing disease condition in the newborns. It can regress completely in some, regress with some sequelae in others while progress to severe retinal detachment and vision loss in a few babies. This complex and variably progressive nature of ROP warrants a robust description of the disease and its classification into various severities, which helps clinicians to properly document, prognosticate and treat the disease.

In 1979, an international committee involving 23 ophthalmologists from 11 countries was formulated and the “International Classification for Retinopathy of Prematurity” (ICROP) was devised.^2^

ICROP 1984 and a modification in 1987 takes into account three major aspects of the disease – its location, extent and severity.

**Figure 1a:**
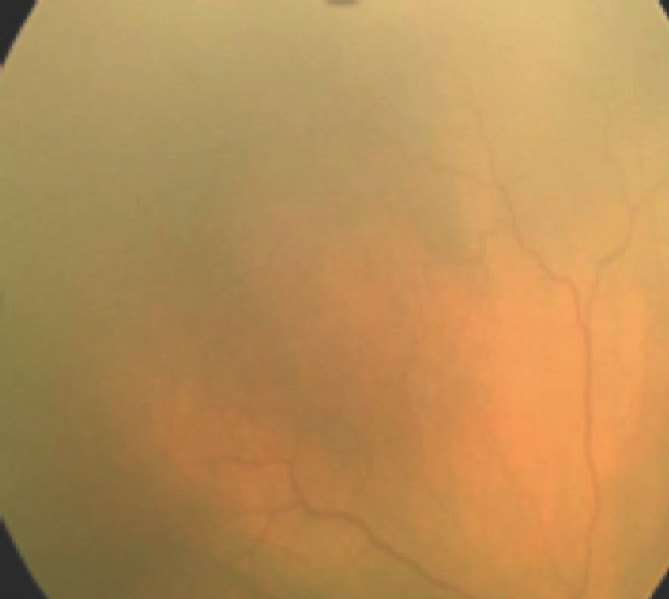
Immature retina with dichotomous branching and

**Figure 1b:**
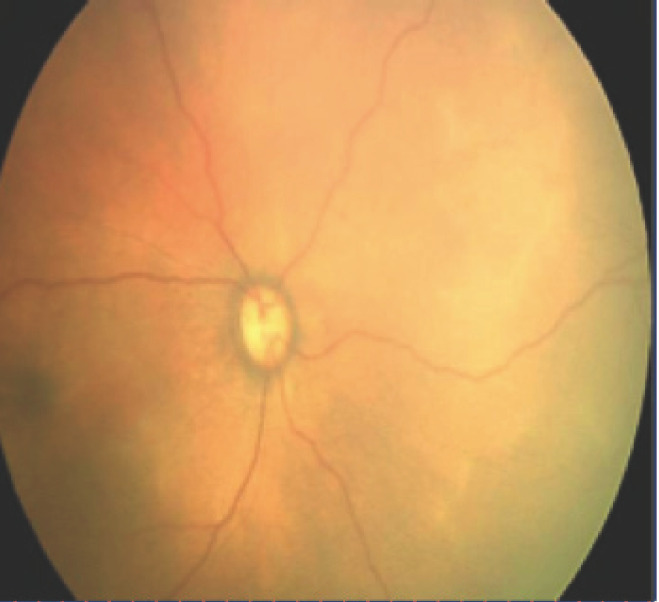
Posterior pole with normal vessel caliber in a premature baby

## Location

**Figure 2:**
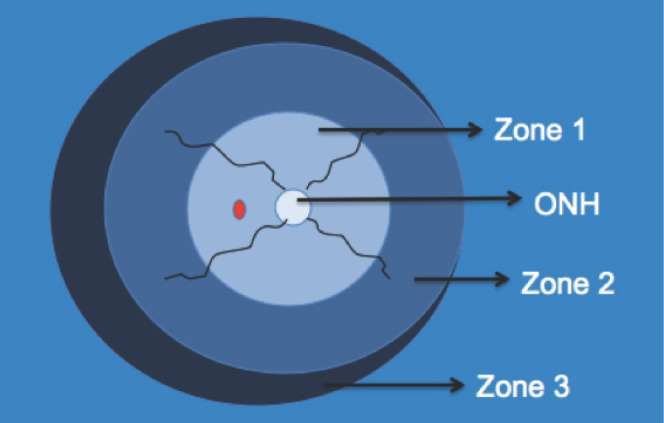
Retina divided into 3 zones to describe the location of ROP

The retina is divided into three zones centered on the optic disc (Figure 2). More posterior the disease, more severe and likely the progression is seen. Zone 1 is defined as the circle, the radius of which is twice the distance between the center of optic disc and center of macula. Zone 2 is defined as the area from the edge of the zone 1 peripherally to a point tangential to the nasal ora serrata. Zone 3 is the residual temporal crescent of retina anterior to zone 2.^2^

### Extent

Extent of ROP is defined by the hours of the clock from 1–12 with each clock hour at 30 degrees.^2^

### Severity

The disease is staged according to severity, in four stages. It was also realised that the disease can exist in more than one stage in the eye at a time. For staging, the worse stage was noted, however, for proper documentation, it was recommended that the extent of each stage should be defined in clock hours.^2^

## Stage 1, demarcation line

A thin delicate line-like white structure separating the vascular and avascular retina is visible. There is abnormal branching and arcading leading to it. It is relatively flat and lies in the plane of the retina.

## Stage 2, ridge

It is a line which has grown and has a volume of thickness and height. It extends above the plane of the retina. Small tufts of new vessels may be found

## Stage 3, extraretinal fibrovascular proliferation

This stage is reached when the component of extraretinal fibrovascular proliferation which is continuous with the posterior border of the ridge appears. It grows into the vitreous perpendicular to the ridge.

## Stage 4, retinal detachment

When the fibrovascular proliferation leads to a retinal detachment, it is classified as stage 4. It is often tractional and sometimes exudative.

## Plus disease

Progressive vascular incompetence presenting as dilatation and tortuosity of vessels in four quadrants at posterior pole, iris vascular engorgement leading to pupillary rigidity and vitreous haze comprises the active and progressive status of ROP and is termed as the plus disease.^2^

### Prethreshold and threshold ROP

Threshold ROP was defined as a condition with 50% risk of retinal detachment if left untreated. This includes ROP of more than 5 contiguous or 8 cumulative clock hours of stage 3 plus ROP in zone 1 or zone 2. All eyes with threshold disease were recommended to be treated.

Prethreshold ROP was defined as any zone 1 ROP less than threshold, zone 2 stage 2 with plus, zone 2 stage 3 without plus or zone 2 stage 3 with plus but less than 5 contiguous or less than 8 cumulative clock hours of ROP. Initial recommendations advised followup of these eyes.

Three major problems were encountered while using the ICROP classification of 1984. The first one was the anatomical delineation of zone 1. Anatomical landmarks are ill-defined in premature eyes and hence the divisions of the zones were arbitrary. Secondly, it was also recognised that there was a need to further classify stage 3 due to its prognostic importance. Tractional detachments were classified as stage 4. However, the cicatricial (fibrous scar) forms of the ROP continuum were not classified in the ICROP classification. A revised ICROP classification was put forward in 2005^3^ by a committee of 15 ophthalmologists. This new classification tried to cover the gaps of the previous one with the new insights provided by the upgraded imaging technologies for prematures.^4^

Three main highlights of the revised system were:

- Description of an aggressive form of ROP in babies with very low birth weight– aggressive posterior ROP (APROP).
- Recognition of a “pre-plus” form of the disease, intermediate to the normal vessels and plus disease as described earlier.
- Anatomical definition of zone 1.

The revised ICROP classification of 2005^3^ retained descriptions under the three major aspects of the original classification: location, extent and severity.

### Location

The zones were defined as in the earlier classification. For better understanding during practical use, it was recommended to use a 25 or 28D lens with the optic disc at the nasal edge. The image formed was described as zone 1.

### Extent

Extent of the disease was described in clock hours as per the original classification.

### Severity

The revised classification^3^ divided ROP into five stages.

### Stage 1, demarcation line

Same as ICROP 1984.

### Stage 2, ridge

Same as ICROP 1984.

### Stage 3, extraretinal fibrovascular proliferation

Same as ICROP 1984. In addition proliferation was further divided into mild, moderate and severe.

**Figure 3a:**
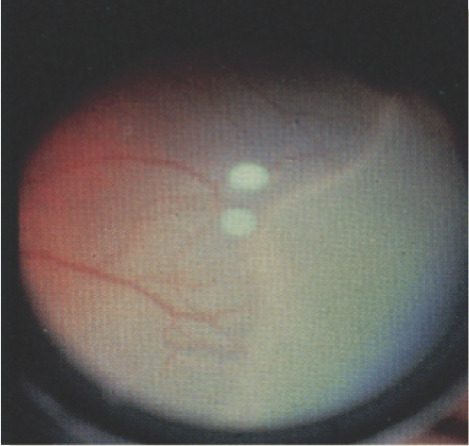
Stage 1, demarcation line

**Figure 3b:**
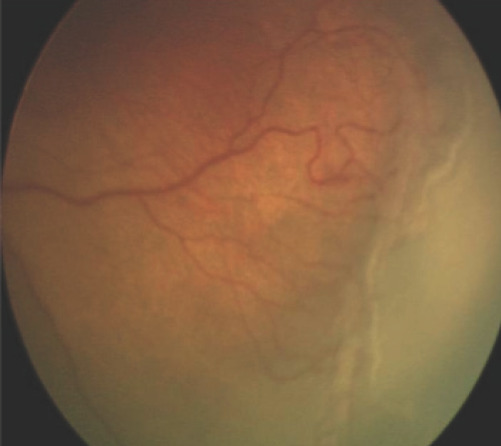
Stage 2, ridge

**Figure 3c:**
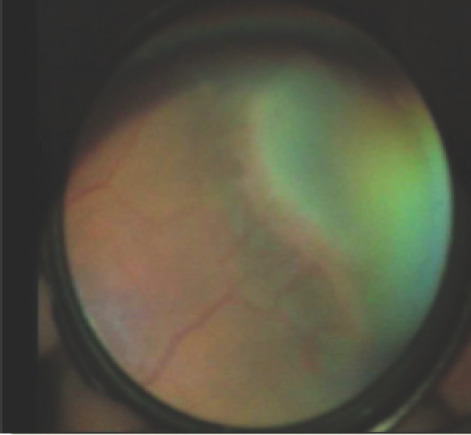
Stage 3, extraretinal proliferation

**Figure 3d:**
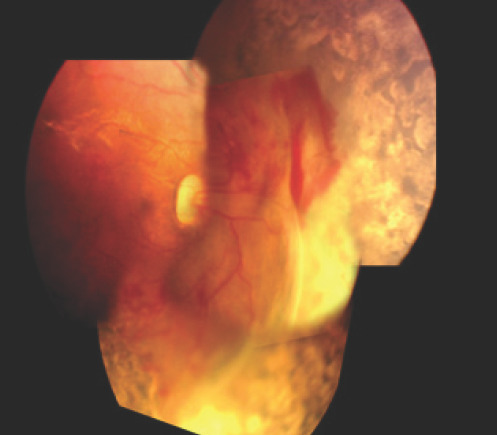
Stage 4A ROP, post laser partial retinal detachment, not involving fovea

### Stage 4, partial retinal detachment

The 2015 revision classifies the tractional retinal detachments into extrafoveal (Stage 4A, Figure 3d) and foveal (Stage 4B, Figure 3e). They are usually circumferentially oriented and described according to the clock hours involved.

**Figure 3e:**
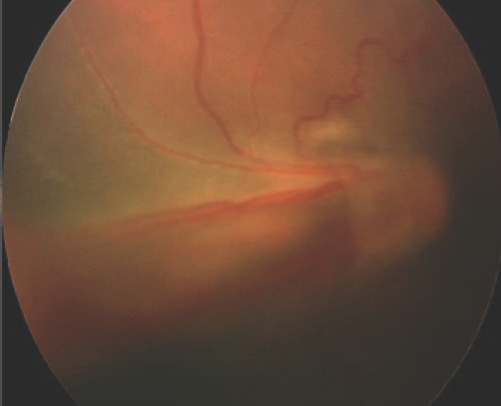
Stage 4B, partial retinal detachment involving fovea

**Figure 3f:**
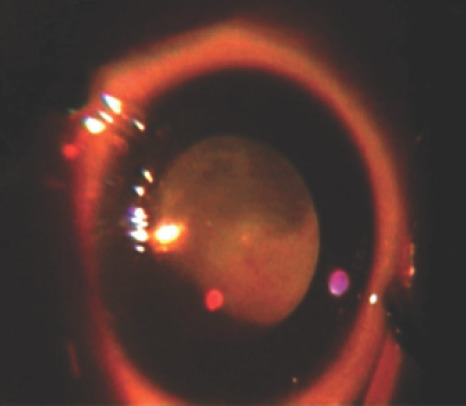
Stage 5, total retinal detachment

### Stage 5, Total retinal detachment

They are funnel shaped and mostly tractional in nature (Figure 3f).

#### The concept of “pre-plus disease”

**Figure 4:**
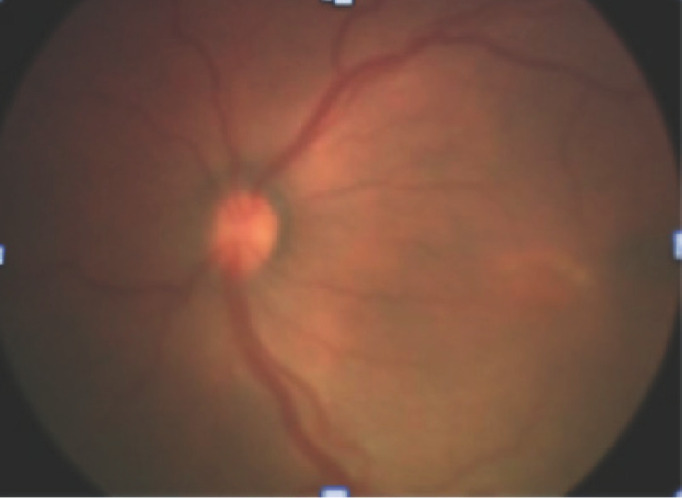
Pre-plus disease

The revised ICROP classification recognised and defined the state of active ROP where the features were insufficient for the diagnosis of plus disease but the vascular changes were more marked than normal. This entity was called “pre-plus disease” (Figure 4). This signified the pre stage which could in further course of time develop into plus disease.

#### Plus disease

**Figure 5:**
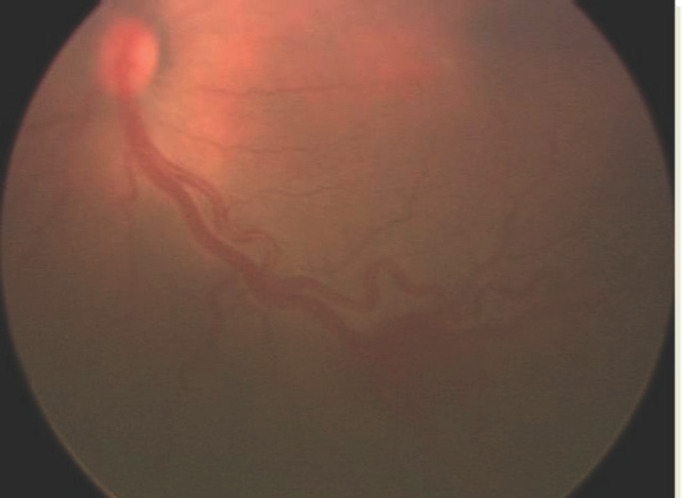
Plus disease showing dilatation and tortuosity of posterior pole vessels

Increased venous dilatation and arteriolar tortuosity of posterior vasculature, with increasing iris engorgement, pupillary rigidity and vitreous haze were defined under the more active ROP, “plus disease”. A standard clinical photograph (Figure 5) was used to define the disease. At least two quadrant involvements of the signs were required to define the disease as plus disease. This was a change from the original four quadrants.

#### Aggressive posterior ROP

A rapidly progressive, ill-defined form of ROP had been previously described as type II ROP or “Rush disease” or “Fulminate ROP”. It was not specifically included in the original ICROP classification. The revised classification defines it as the “aggressive posterior ROP” (Figure 6a). It is characterised by severe dilatation and tortuosity of the vessels which is out of proportion to the peripheral retinopathy. The disease is limited to the posterior pole in zone 1 or posterior zone 2 and usually does not progress through the classic stages 1–3 of ROP.

**Figure 6a:**
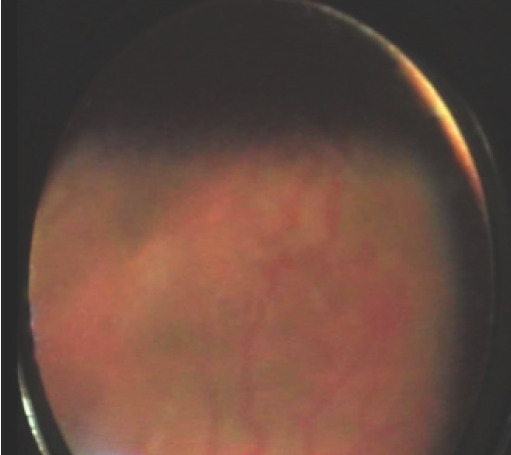
APROP with avascular pockets and shunts

**Figure 6b:**
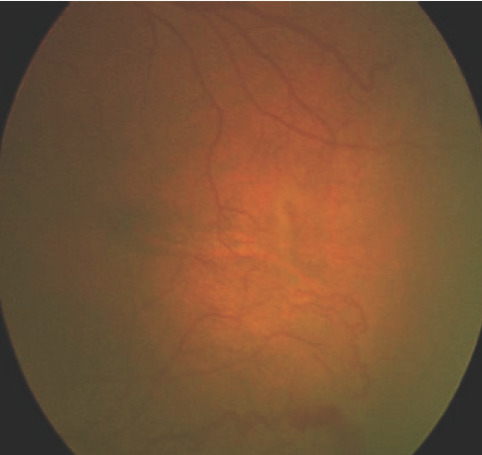
Hybrid ROP with shunting of vessels.

Shunting occurs between vessels intraretinally and flat neovascularisation is noted. It extends circumferentially and if left untreated, very rapidly progresses to Stage 5 within few days. This type of ROP can be easily missed.

### Staged ROP versus APROP

It is very important to distinguish these two. Some important differences are in Table 1.

## Current guidelines for treatment

In 2005, randomised trials of early stages of ROP showed better outcomes than treating at threshold ROP stage. Prethreshold ROP was divided into two types.^4^

### Type 1 high risk prethreshold ROP

Defined as zone 1 plus with any stage, zone 1 stage 3 with no plus and zone 2 stage 2 or 3 plus. All eyes with type 1 prethreshold ROP are currently recommended for immediate treatment.

### Type 2 low risk prethreshold ROP

Defined as zone 1 stage 1 or 2 without plus disease and zone 2 stage 3 without plus disease and follow-up is recommended for such eyes.

### Cicatricial ROP

Untreated or partially treated eyes can present with cicatricial ROP with sequalae as disc and macular dragging, peripheral vitreoretinal-lenticular adhesions and subretinal pigmentation from reattached exudative detachments. These eyes have variable potential for vision and often benefit from low vision services.

## Future directions

Several studies have compared the interobserver agreement of diagnosing various stages of ROP.^5–7^ It has been noted well that while the agreement on diagnosing the treatment requiring stages of ROP is good, the earlier stages of ROP have discrepancies. This has led us to believe that other objective features need to be added in the classification to increase the interobserver agreement especially with telemedicine being increasingly used in the screening of ROP.

Klufas et al used fundus fluorescein angiography (FFA) in addition to color fundus photographs and noted that the agreement on the diagnosis improved significantly with the objective assessment of FFA.^8^ Although FFA alone didn't show any significant advantage, defining characteristics on the newer imaging techniques in today's era would probably be beneficial and overcome the drawbacks of existing classification.

Newer disease presentations have been described such as ‘hybrid ROP’^9^ that has components of both staged ROP and APROP (Figure 6b). Other gaps in ROP classification include absence of classifying regressing new vessels in ROP, regression of plus stages to post plus states, classifying the rare exudative or rhegmatogenous presentations, classifying progressive stage 5 ROP, identifying various severities of evolving APROP, and classifying the disease based on possible differences in the pathogenesis of staged ROP and APROP.

**Table 1:** Staged ROP versus APROP

	Staged ROP	Aggressive Posterior ROP
Pattern of vessels	Dichotomously branching	Looping and shunting
Plus and preplus	Clearly made out	Very subtle and appears suddenly
Location of new vessels	Appear at junction of vascular and avascular retina, often temporally	Appear at any place, especially can start nasally as well
Type of new vessels	As individual twigs growing vertically into vitreous	Flat new vessels with each vessel ill defined and almost like a globule of vessels
Junction	Avascular and vascular junction are very well defined and often ‘wavy’ and continuous	No definitive junction as multiple pockets of avascular retinal tissue are enclosed within vascularised boundary. Pockets are discontinuous.
Location	In any zone	In zone I or posterior zone II
Progression timeline	Progresses to detachment over 3–6 weeks or more going through each stage for variable time periods	Progresses to detachment within few days and may not show each stage clearly
Progression pattern	Progresses through each stage with each stage lasting at least for 1–2 weeks	Early phases of APROP are not classified well as yet. Each phase may be lasting for only short time
Response to timely treatment	Excellent response in most cases	May respond poorly and treatment failures can occur
Detection of disease	Not difficult as findings are clear	Often missed as findings are unclear
